# Epicatechin Decreases *UCP2* Gene Expression in MDA-MB-231 Breast Cancer Cells by the Presence of a Regulatory Element in the Promoter

**DOI:** 10.3390/ijms26094102

**Published:** 2025-04-25

**Authors:** Fernando Pereyra-Vergara, Ivonne María Olivares-Corichi, Juan Pedro Luna-Arias, David Méndez-Luna, José Rubén García-Sánchez

**Affiliations:** 1 Sección de Estudios de Posgrado e Investigación, Escuela Superior de Medicina del Instituto Politécnico Nacional, Ciudad de México C.P. 11340, Mexico; pereyravfer@gmail.com; 2 Departamento de Biología Celular, Centro de Investigación y de Estudios Avanzados del Instituto Politécnico Nacional (Cinvestav-IPN), Ciudad de México C.P. 07360, Mexico; jpluna@cinvestav.mx; 3 Departamento de Fisiología, Escuela Nacional de Ciencias Biológicas, Instituto Politécnico Nacional, Zacatenco, Av. Wilfrido Massieu 399, Col. Nueva Industrial Vallejo, Alcaldía Gustavo A. Madero, Ciudad de México C.P. 07738, Mexico; meld8909@gmail.com

**Keywords:** transcriptional regulation, triple negative breast cancer, zinc finger proteins, *UCP2* promoter

## Abstract

Uncoupling protein 2 (UCP2) plays an important role in normal cells because it mitigates the cytotoxic effect of reactive oxygen species (ROS). However, its overexpression in cancer cells is related to drug resistance and increased cell proliferation due to a decrease in ROS production. In this context, molecules that regulate or block UCP2 have potential as anticancer agents. (-)-Epicatechin, a flavonoid that inhibits cell proliferation, increases ROS, and induces apoptosis in cancerous cells, was evaluated for its effects on *UCP2* gene expression. For this purpose, the real-time quantitative polymerase chain reaction (qRT–PCR) and Western blotting were performed in MDA-MB-231 and MCF-10A cells to determine the effects of (-)-epicatechin on UCP2 expression. Furthermore, the impact of (-)-epicatechin on cell viability was also determined. To analyze the transcriptional regulation of the *UCP2* gene by (-)-epicatechin, a 5′-region of the human *UCP2* gene (−2093/+297) was amplified, sequenced, cloned, and inserted into a reporter plasmid. To analyze the promoter activity and regulatory motif involved in the effects of (-)-epicatechin, several deletions of the *UCP2* promoter were generated and transfected into MDA-MB-231 and MCF-10A cells. An electrophoretic mobility shift assay (EMSA) was carried out to detect the interaction between DNA and proteins involved in the effect of (-)-epicatechin. The increased expression of the *UCP2* gene in MDA-MB-231 cells was decreased by (-)-epicatechin, and the opposite effect was observed in MCF-10A cells. The promoter region of the human *UCP2* gene (−2093/+297) showed activity, which was decreased by (-)-epicatechin. A sequence of 117 bp located at position −109 b to +8 b has a fragment of 90 bp that is related to the (-)-epicatechin effect. Bioinformatics analysis and EMSA of this sequence revealed the presence of a regulatory site for a protein with zinc fingers. The presence of a response element to (-)-epicatechin in the human *UCP2* promoter revealed that the inhibition of this gene in MDA-MB-231 breast cancer cells occurred at the transcriptional level. In this study, we propose the mechanism of action of (-)-epicatechin that could aid in cancer treatment.

## 1. Introduction

Uncoupling proteins (UCPs) are a family of anion transporters located in the inner membrane of mitochondria that transfer hydrogen ions from the inner membrane to the matrix [1]. This proton flow decouples oxidative phosphorylation by reducing the mitochondrial membrane potential, resulting in the conversion of energy (heat dissipation) without ATP production [1]. In mammals, five UCPs have been identified (UCP1–5) [2]. UCP1 was called “thermogenin” due to its nonshivering thermogenesis [3]; however, it was renamed as uncoupling protein because it bypasses complex V of the electron transport chain, returning protons to the matrix and releasing the Gibbs free energy stored in protonmotive force as heat [3]. UCP1 has 59% homology with UCP2 and 3 [4]. In humans, UCP2 is expressed throughout the body, and the main function is the regulation of reactive oxygen species production (ROS) and protection against oxidative damage [5]. UCP3 shares 73% homology with UCP2, and investigation into its function revealed that it prevents oxidative damage by decreasing ROS [6]. UCP4 and UCP5 are less identical to UCP1–3 [7]; they are predominantly expressed in the central nervous system of humans [8,9] and have a neuroprotective effect against oxidative damage [8]. UCPs have an important influence on mitochondrial bioenergetics, thermogenesis and control of ROS production, so if they become impaired, several disorders can develop [1]. One of the most studied is UCP2, which is associated with different malignant tumors, including breast cancer [10,11,12]. Several studies have investigated UCP2 function in cancer; however, conflicting data have been reported. Some studies suggest that UCP2 promotes tumor progression by promoting aerobic glycolysis (Warburg effect) and inhibiting the generation of reactive oxygen species (ROS) [10,13]. Alternatively, UCP2 can also inhibit the proliferation of cancer cells by increasing ROS production [14], reprogramming cellular metabolism [15], or enhancing immunity against cancer [16]. In addition, UCP2 expression is associated with tumor grade and chemoresistance [17,18]. These findings indicate that UCP2 expression plays an important role in cancer.

UCPs are known to have strict transcriptional regulation; indeed, models in rats and mice have been used to establish expression tissue-specificity, its hormonal regulation, and the detection of cis-/trans-acting elements in the promoter regions of UCP genes [19,20,21]. However, no studies on human UCP2 regulation or descriptions of molecules that interact with its promoter and modify its expression exist.

Flavonoids are molecules of natural origin with a phenolic structure and are found in plants [22]. Seven subclasses of flavonoids have been described: flavones, flavanones, flavanols, flavonols, isoflavones, anthocyanidins and chalcones [23]. Flavonoids have several biological properties, including anticancer properties [24]; indeed, the mechanisms involved include cell cycle arrest, proliferation inhibition, apoptosis, antioxidant activity and antimetastatic effects [25,26]. In this context, we have demonstrated that the flavonoid (-)-epicatechin and its procyanidins have an antiproliferative effect on MDA-MB-231 breast cancer cells [27,28], which is related to the induction of apoptosis and ROS generation [27]. This evidence strongly suggests the possible regulation of the UCP2 protein by (-)-epicatechin. In this study, two breast cell lines (MDA-MB-231 breast cancer cells and MFC10A noncancerous breast cells) were used to determine the possible regulation of UCP2 protein expression by (-)-epicatechin and its relationship with the resulting antiproliferative effect. In addition, we established the region of the *UCP2* promoter that is involved in this regulation.

## 2. Results

### 2.1. Differential UCP2 Expression Among MDA-MB-231 Breast Cancer Cells and MCF10A Cells (Breast Noncancerous Cells)

**Figure 1:**
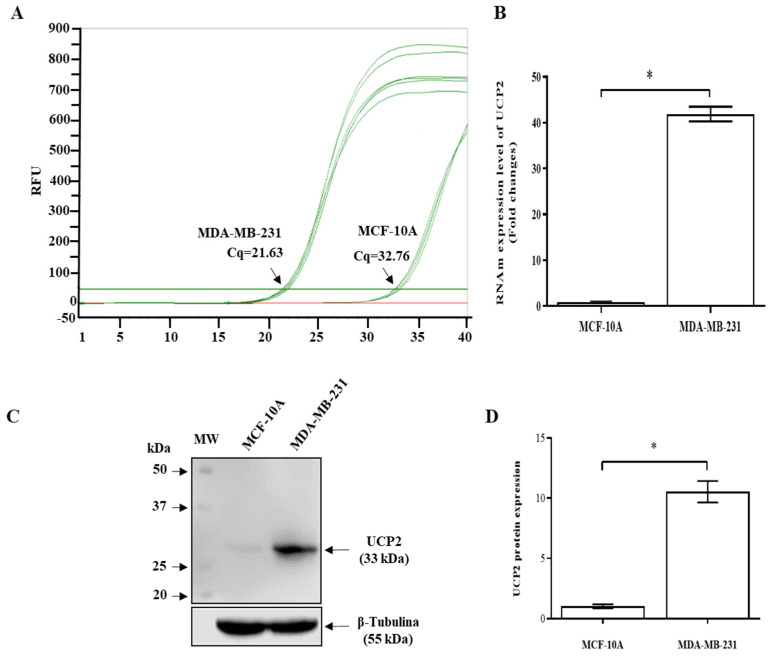
Expression of the human *UCP2* gene in MDA-MB-231 and MCF-10A cells. (**A**) Amplification plots of qRT–PCR analysis for the expression of UCP2 in MDA-MB-231 and MCF-10A cells. (**B**) Comparative analysis of the RNA expression of UCP2 in MCF10A and MDA-MB-231 cells. (**C**) The expression level of UCP2 was analyzed via Western blotting. (**D**) Comparative analysis of UCP2 protein expression in MCF10A and MDA-MB-231 cells. The data are expressed as the means ± SD of three independent experiments and were analyzed by Student’s *t*-test (* *p* < 0.001).

In this work, we investigated the role of UCP2 in the cellular death induced by flavonoids. The expression of the *UCP2* gene was analyzed at the RNA and protein levels. Figure 1A shows the qRT-PCR amplification plots generated by human UCP2 primers and using a cDNA template from RNA obtained from MDA-MB-231 and MCF10A cells (breast noncancerous cells). The plots revealed that the qRT-PCRs started with a smaller number of cycles in the MDA-MB-231 cells than in the MCF10A cells (Figure 1A). Indeed, the expression of UCP2 mRNA in MDA-MB-231 cells was 44.4-fold greater than that in MCF10A cells (Figure 1B).

This difference in mRNA expression was also observed at the protein level (Figure 1C). These data revealed high expression of UCP2 in MDA-MB231 breast cancer cells (10-fold higher) than in MCF10A cells (Figure 1D).

### 2.2. Treatment with (-)-Epicatechin Decreased UCP2 Expression in MDA-MB-231 Cells but Not in MCF10A Cells

**Figure 2:**
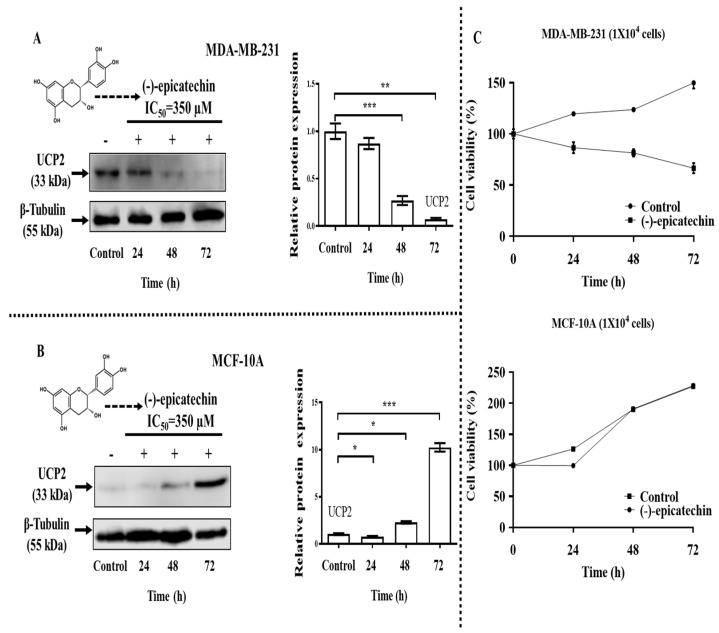
Effect of (-)-epicatechin on *UCP2* gene expression and growth of cell lines. (**A**) MDA-MB-231 and (**B**) MCF-10A cells were treated with (-)-epicatechin (350 µM) using methanol (vehicle) as a control, and *UCP2* gene expression was analyzed at the indicated times via Western blotting. The data are expressed as the mean ± SD of three independent experiments. *, **, *** *p* < 0.001. (**C**) Effect of (-)-epicatechin on cell viability. Both cell lines were treated with (-)-epicatechin (IC_50_ = 350 µM), and cell viability was measured at the indicated times via a [3-(4,5-dimethyl-2-thiazolyl)-2,5-diphenyl-2H-tetrazolium bromide] (MTT) assay. Each data point was analyzed in triplicate in three independent experiments and is presented as the mean ± SD.

To establish *UCP2* expression in both cell lines, we investigated the effect of (-)-epicatechin on *UCP2* expression. The treatment of MDA-MB-231 breast cancer cells with flavonoids (IC_50_ = 350 μM) resulted in a decrease in *UCP2* gene expression, which was observed from 48 to 72 h of incubation with the flavonoid (Figure 2A). In contrast, in MCF10A cells (noncancerous cells), flavonoids increased the protein expression of UCP2 at the same time (48 to 72 h) (Figure 2B).

To establish the relationship between changes in UCP2 protein expression and cell viability, we grew both cell lines in the presence of flavonoids (IC_50_ = 350 µM), and the effects of flavonoids on cell viability were determined. Compared with the control, the flavonoid decreased the viability of MDA-MB-231 cells from 24 to 72 h of incubation (Figure 2C); moreover, in MCF-10A cells, no effect was observed (Figure 2C). These data demonstrated the inverse relationship between UCP2 protein expression and the viability of MDA-MB-231 breast cancer cells.

### 2.3. Transcriptional Regulation of the Human UCP2 Gene by (-)-Epicatechin

To investigate whether the *UCP2* gene is transcriptionally regulated by (-)-epicatechin, a 2390 bp segment of the promoter region of the *UCP2* gene was amplified via PCR, cloned and sequenced (Supplementary Data S1).

**Figure 3:**
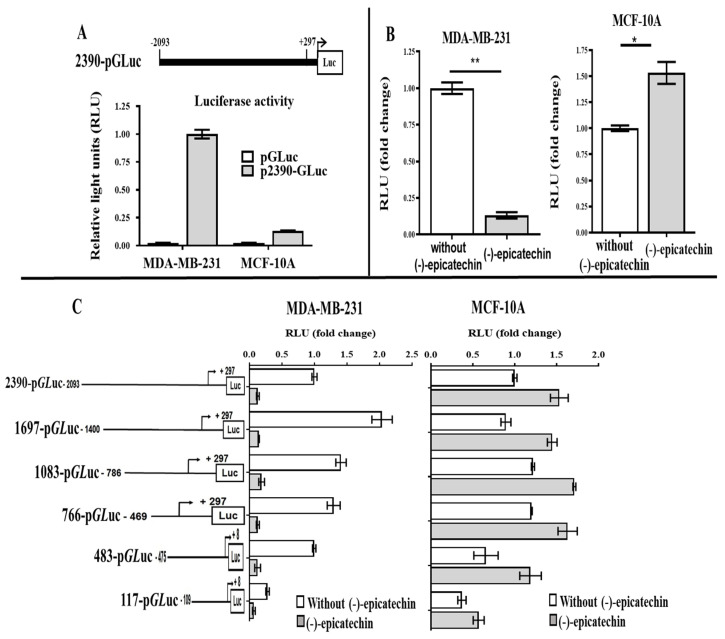
Activity of the *UCP2* promoter and various lengths of the 5′-flanking region cloned from MDA-MB-231 and MCF-10A cells. (**A**) Schematic representation of the *UCP2* promoter cloned in pGLuc plasmids (2390-pGLuc) and its basal activity. (**B**) Relative luciferase activity of the 2390-pGLuc plasmid, *, ** *p* < 0.05 and (**C**) pGLuc plasmids containing various lengths of the 5′-flanking region cloned in the presence or absence of (-)-epicatechin. The data are presented as the mean ± SD of three independent experiments.

Blast analysis of the sequence revealed 100% homology with the −2093/+297 region of the *UCP2* gene [Homo sapiens (human)] (GenBank NG_011478.1). To determine the transcriptional activity of the cloned region, the sequence was subcloned and inserted into the pGLuc-Basic 2 vector to obtain a pUCP2 (−2093 to +297)-luciferase fusion construct (renamed p2390-GLuc) (Figure 3A). This construct was transfected into MDA-MB-231 and MCF10A cells, after which the luciferase activity was determined. The data revealed that the 2390 bp fragment generated a greater luciferase expression level in MDA-MB-231 cells than in MCF10A cells (Figure 3A).

The activity of the p2390-GLuc construct was analyzed in the presence of flavonoids because the decrease in UCP2 by (-)-epicatechin could involve regulation through the promoter. The p2390-GLuc construct was transfected into the MDA-MB-231 and MCF-10A cell lines, which were then treated with (-)-epicatechin. In MDA-MB-231 cells, the flavonoid decreased the luciferase activity by 87% (Figure 3B). On the other hand, in MCF-10A cells, the activity increased by 53% in the presence of the flavonoid (Figure 3B). These data indicated that (-)-epicatechin has a transcriptional effect and that the sequence involved is present in this 2390 bp region.

To determine the promoter region involved in the regulation of UCP2 by flavonoids, five constructs with lengths of 1697 bp, 1083 bp, 766 bp, 483 bp and 117 bp bound to GLuc were generated and analyzed in the presence of the flavonoid. Figure 3C shows a schematic representation of the constructs generated and the activity of luciferase in both cell lines. Interestingly, in the MDA-MB-231 cells, all the constructs presented a decrease in luciferase activity in the presence of (-)-epicatechin (Figure 3C); however, in the MFC-10A cells, an increase was observed (Figure 3C). We postulated that a response element found in these 117 nucleotides was involved in these effects because the construction of 117-pGLuc (lengths −109 to +8) was shorter and showed the same behavior as the other construct in both cell lines (in MDA-MB-231 and an increase in MCF-10A) (Figure 3C).

**Figure 4:**
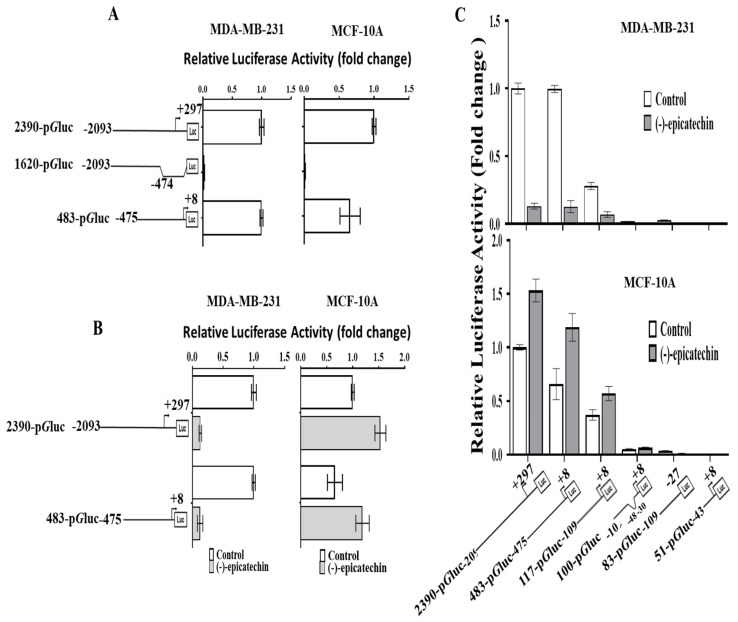
A 5′ region of 117 bp contains the response element for (-)-epicatechin. (**A**) Comparison of the promoter activity of 2390-pGLuc with those of 1620-pGLuc (without) and 483-pGLuc (with) in response to (-)-epicatechin. (**B**) Similar promoter activity of 2390-pGLuc versus 483-pGLuc in the presence of (-)-epicatechin (decrease in MDA-MB-231 cells or increase in MCF-10A cells). (**C**) Deletions and promoter activity of 483-pGLuc. The 117-pGLuc construct is the shortest sequence that affects (-)-epicatechin in both cell lines. The data are shown as the mean ± SD of three independent experiments.

To test this hypothesis, the activity of the complete sequence (2390-pGLuc) was compared with that of the 1620-pGLuc construct (−2390 to −474) and 483-pGLuc construct (−475 to +8). Interestingly, the 1620-pGLuc construct (containing more than 770 bp of the 5′ region, including the 117 bp in the studied area) lost luciferase activity (Figure 4A). In contrast, the construct 483-pGLuc (which included 117 bp in the studied area) showed the same activity as the complete construct (2390-pGLuc) (Figure 4A); this behavior was observed in both cell lines. These data corroborate the presence of important sequences, including the sequence 117 bp in length (−109 bp and +8 bp), which are involved in the activity of the promoter. In addition, when these constructs were assayed in the presence of (-)-epicatechin, a decrease (MDA-MB-231) or increase (MCF-10A) in expression was observed (Figure 4B).

### 2.4. The 5′ Region of 117 Bp Is a Minimal Promoter Region That Contains the Elements That Respond to (-)-Epicatechin and the Basal Promoter Activity of the UCP2 Gene

Considering that the activity of 2390-pGLuc was the same as that of 483-pGLuc (Figure 4A), the latter was used to generate the 117-pGLuc construct (−109 to +8). The luciferase activity of the 117-pGLuc construct was less than those of 2390-pGLuc and 483-pGLuc (Figure 4C); however, the (-)-epicatechin effect on 117-pGLuc was observed in both cell lines (Figure 4C). Finally, when specific deletions were performed on pGLuc-117 to generate 100, 83 and 51 bp pGLuc, these constructs resulted in the loss of promoter activity (Figure 4C). These data indicated that the region of 117 bp (−109 to +8) contains the response element for (-)-epicatechin and the elements that maintain the minimal activity of the promoter.

**Figure 5:**
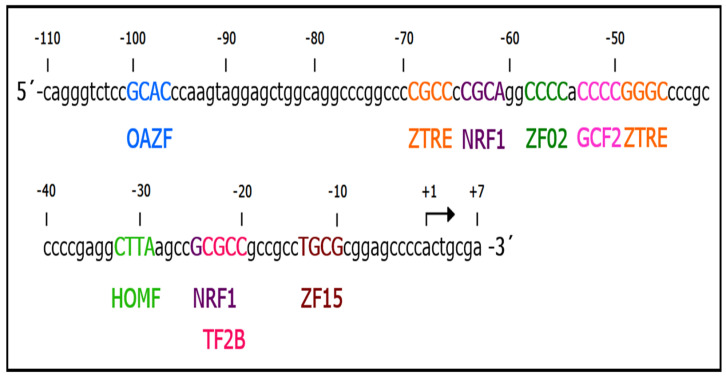
Schematic representation of possible response elements in the minimal region promoter of the *UCP2* gene (117 bp). The nucleotide sequence, the core of elements and the potential transcription factor are shown. Nucleotides are numbered considering the transcription initiation site (+1).

The deletions of the pGLuc-117 construct did not delimit the sequence involved in the (-)-epicatechin response; therefore, an in silico analysis was performed to identify possible response elements inside this minimal region promoter. We used the software Matinspector tool (release 8.4.1) on the Genomatix software suite v3.11 (Intrexon Bioinformatics Germany GmbH, Munich, Germany) and possible response elements were detected (Figure 5). Ten possible response elements were observed; interestingly, five were related to zinc finger proteins, two elements were related to nuclear respiratory factor 1 (NRF1), an element for GC-binding factor 2 (GCF2), a repressor of EGFR, an element for homeodomain transcription factors (HOMF) whose function is essential in the development of adult organisms, and an element for RNA polymerase II transcription factor II B (TF2B), which positions TFIIB on the DNA in the proper orientation for interactions with Pol II (Figure 5 and Supplementary Data S2).

### 2.5. One Element in the 5′ Region Is Required for the Regulatory Effect of (-)-Epicatechin on UCP2 Expression

**Figure 6:**
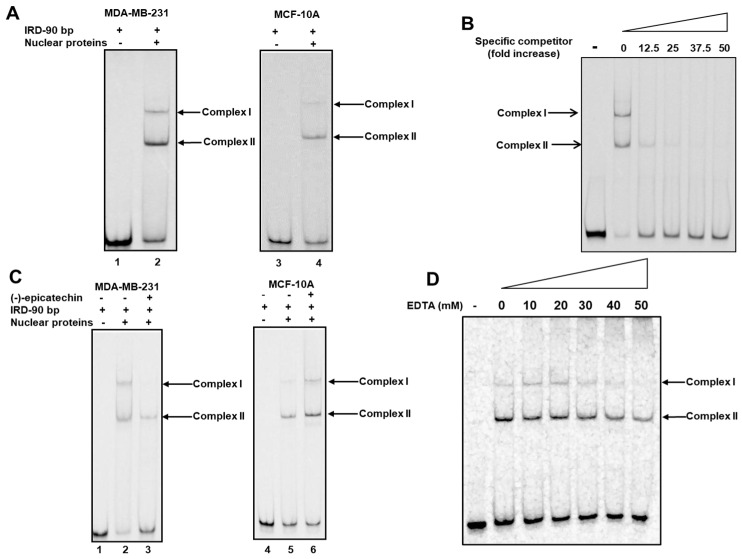
A segment of 90 bp (−82/+8 region) is required for the (-)-epicatechin effect. (**A**) Binding activity was determined via EMSA. Labeled oligonucleotides of 90 bp (IRD-90 bp, 25 fmol) were incubated without (lanes 1 and 3) and with (lanes 2 and 4) nuclear protein from MDA-MB-231 and MCF-10A cells. The generation of two complexes was observed (lanes 2 and 4). (**B**) IRD-90 bp was incubated with nuclear protein from MDA-MB-231 cells in the presence of an excess of 90 bp nonlabeled oligonucleotide, showing the specificity of the complexes. (**C**) (-)-Epicatechin inhibits complex I formation. Lanes 1 and 4 IRD-90 bp in the absence of nuclear protein. IRD-90 bp was incubated with nuclear protein from untreated MADA-MB-231 and MCF-10A cells (Lanes 2 and 5) and those treated with (-)-epicatechin (Lanes 3 and 6). (**D**) Magnesium inhibits complex I formation. IRD-90 bp was incubated with nuclear protein from MDA-MB-231 cells in the presence of different EDTA concentrations.

With these data, we analyzed the presence or absence of an interaction between DNA and proteins. A segment of 90 bp (−83/+7 region) from the sequence of 117 bp was used in electrophoretic mobility shift assays (EMSAs) (Figure 5). The nuclear proteins of MDA-MB-231 or MCF10A cells were incubated with infrared fluorescent dye labeled with 90 bp (IRD-90 bp). Interestingly, both nuclear proteins resulted in the formation of two complexes (Figure 6A, lanes 2 and 4); however, complex 1 was less intense than the nuclear protein from MCF10A cells (Figure 6A, lane 4).

The specificity of the generated complex was tested by the addition of different numbers of unlabeled 90 bp oligonucleotides, and the effective competence of the complexes formed by the labeled IRD90 was observed (Figure 6B).

To demonstrate the effect of (-)-epicatechin on the formed complexes, an EMSA with nuclear proteins from cells treated with (-)-epicatechin was performed. Figure 6C shows that the flavonoid inhibited the formation of complex I with nuclear protein from MDA-MB-231 cells (Figure 6C, lane 3), whereas complex I was intensified with nuclear protein from MCF-10A cells (Figure 6C, lane 6). These data indicate that (-)-epicatechin in MDA-MB-231 cells inhibits the binding of a transcription factor, whereas that in MCF-10A cells is favored. These DNA–protein interactions are generated in the presence of flavonoids and are associated with a decrease or increase in the activity of luciferase in MDA-MB-231 and MCF-10A cells, respectively (Figure 3B). We propose that complex I is involved in the regulatory effect of (-)-epicatechin on UCP2 expression.

Considering that two complexes were formed and that two response elements for NRF1 were detected at 90 bp (Figure 5), a supershift assay was performed using an antibody against the NRF1 protein. We observed that preincubation of nuclear protein from MDA-MB-231 cells with the antibody did not result in any electrophoretic changes (Supplementary Data S3). Indeed, we found the same observation when the antibody was incubated with the complex formed (Supplementary Data S3).

We performed EMSA in the presence of different concentrations of EDTA, a potent Zn^2+^ chelating agent, because five elements for zinc finger transcription factors were detected and because Zn^2+^ mediates DNA interactions [29]. Figure 6D shows that incubation with EDTA (10–50 mM) eliminated the formation of complex I but not that of complex II. These data indicate that complex I is formed by a transcription factor with a zinc finger, whereas complex II is generated by another type of transcription factor. Considering that five elements respond to zinc finger proteins and that the C_2_H_2_-zinc finger family is the most numerous and diverse, more studies are necessary to identify the zinc finger protein and response element involved in the transcriptional regulation of the *UCP2* gene by (-)-epicatechin.

## 3. Discussion

The polyphenol (-)-epicatechin, a member of the catechin family (flavan-3-ol), is also known as a flavonoid. (-)-Epicatechin is a molecule with strong antioxidant activity because it possesses many hydroxyl groups [30]. Evidence indicates that (-)-epicatechin has beneficial effects on health, which are believed to be due to its antioxidant and anti-inflammatory properties [31,32,33].

Previously, we described the anticancer activity of (-)-epicatechin, which is selective for cancer cells and is related to the induction of apoptosis [27]. In addition, we reported an increase in reactive oxygen species, suggesting effects at the mitochondrial level. In this study, we demonstrated a downregulation of *UCP2* gene expression by (-)-epicatechin in MDA-MB-231 breast cancer cells, whereas in MCF-10A (noncancerous) cells, the opposite effect was observed.

The UCP2 protein is located on the inner mitochondrial membrane, and its function uncouples the proton gradient in this membrane. Therefore, an accumulation of protons occurs in the intermembranal space; however, ATP synthase facilitates their flow into the matrix to generate ATP [1,34]. Interestingly, when UCP2 is upregulated, the opposite effect occurs (anions and protons leave the matrix), and ATP production through the electron transport chain decreases, resulting in heat-energy release [35,36].

**Figure 7:**
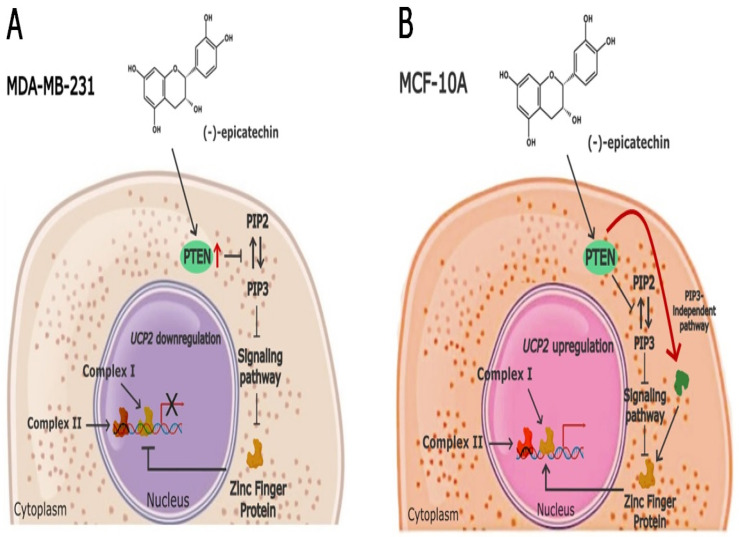
Possible mechanism of the origin of complexes I and II and effects on UCP2 expression. (**A**) (-)-epicatechin could be activating PTEN in MDA-MB-231 cells, which inhibit PIP3-dependent kinases, causing the inactivation of a signal pathway involved in the activation and binding of the transcription factor to the *UCP2* promoter (complex I). (**B**) In MCF-10A, the activation of the PIP3-independent pathway is activating the transcription factor, and it binds to the *UCP2* promoter.

Other functions described for the UCP2 protein include the transport of metabolites for NADPH generation (which is required for cancer cell growth), its participation in maintaining the cellular redox state [37,38,39], and, with an increase in mitochondrial Ca^+2^, resistance to chemotherapeutic and apoptosis evasion [13,40]. For these reasons, the inhibition of UCP2 under pathological conditions such as cancer is highly important. In this study, we show the downregulation of *UCP2* gene expression by (-)-epicatechin in MDA-MB-231 breast cancer cells. However, little is known about the transcriptional control of the human *UCP2* gene. In this study, we cloned 2390 bp of the 5′ region of the human *UCP2* gene in a reporter plasmid (2390-pGLuc), characterized it by deletion and transfection into MDA-MB-231 and MCF10A cells, and demonstrated the presence of a regulatory element involved in the (-)-epicatechin effect on the downregulation of the *UCP2* gene. Interestingly, this regulatory element was inside the minimal region of promoter activity (a region also observed by Tu et al., 1999) [41] that corresponds to a 117 bp (−109–+8) fragment. This region was cloned (117-pGLuc) and transfected into MDA-MB-231 cells, which resulted in a decrease in reporter gene expression in the presence of (-)-epicatechin, whereas the opposite effect was observed in MCF-10A noncancerous cells (Figure 3C and Figure 4C). These data agree with the changes observed in UCP2 protein expression in the presence of flavonoids in both cell lines (Figure 2A,B). In this respect, the evidence indicates the presence of an SP1 binding site in this region (transcription factor with zinc fingers) [41]. However, the analysis of possible response elements in this region indicated the presence of five motifs for binding proteins with zinc fingers (Supplementary Data S2). In this context, the data obtained from the EMSA corroborated the participation of a transcription factor with a zinc finger (Figure 6D). In MDA-MB-231 cells, the binding of this transcription factor is inhibited by (-)-epicatechin, resulting in decreased *UCP2* gene expression (in the absence of a transcriptional activator) (Figure 6C, lane 3). The opposite effect was observed in MCF-10A cells (Figure 6C, lane 6), where (-)-epicatechin could favor binding and, consequently, an increase in *UCP2* gene expression. This evidence strongly suggests that the regulatory effect of (-)-epicatechin in both cell lines involves the same transcription factor (Complex I), and its binding or not binding to regulatory elements increases or does not affect *UCP2* gene expression (Figure 7). In this context, the evidence shows that flavonoids can inhibit signal pathways through the activation of phosphatase and tensin homolog (PTEN) [42,43,44,45]. In MDA-MB-231 cells, we propose that (-)-epicatechin could activate PTEN and inhibit PIP3-dependent kinases. This inhibition causes nonactivation and binding of the transcription factor to the promoter of UCP2 (Complex I) (Figure 7). On the other hand, in MCF-10A cells (considered normal), (-)-epicatechin generates the same effect; however, another PIP3-independent pathway could be activating the transcription factor and its binding to the *UCP2* promoter (Figure 7) [46,47]. Resolving the existence of this signaling pathway in normal cells and its dysfunction in cancer cells is an important area for future work. With respect to complex II, its generation could be related to the basal activity of the promoter; however, it is not affected by the treatment with (-)-epicatechin.

Considering that the regulation of *UCP2* expression plays an important role in pathological conditions such as cancer, the search for molecules that inhibit its expression could be a strategy for the treatment of this pathology.

In this context, evidence shows that purine nucleotides inhibit *UCP2* expression under physiological conditions; however, their impermeability makes them nonfunctional [48]. Genipin, a protein cross-linking agent extracted from gardenia fruits, is another molecule that inhibits the *UCP2* protein; indeed, this inhibition occurs through binding to arginine residues of UCP2 [49]. Furthermore, the inhibition of *UCP2* by siRNA has also been studied [50]. In this study, we report the inhibition of *UCP2* gene expression by (-)-epicatechin. To our knowledge, this study is the first to report the regulation of the expression of the *UCP2* gene by a flavonoid. Considering that UCP2 overexpression has been implicated in a mechanism of protection against reactive oxygen species, evasion of apoptosis and drug resistance in cancerous cells, its inhibition with (-)-epicatechin shows high potential as a flavonoid therapeutic. In addition, the specificity of this flavonoid to cancer cells and the absence of a toxic effect of this molecule suggest its use without affecting normal cells.

In conclusion, *UCP2* overexpression was detected in MDA-MB-231 breast cancer cells compared with MCF10A cells (noncancerous cells). This *UCP2* overexpression was decreased by (-)-epicatechin, a flavonoid that shows anticancer activity. This study provides evidence that the *UCP2* gene is regulated by (-)-epicatechin at the transcriptional level. Studies are being conducted to establish the specific sequence inside the 117 bp involved in the effect of (-)-epicatechin, zinc finger protein identification and signal pathway activation.

## 4. Materials and Methods

### 4.1. Cell Culture and (-)-Epicatechin Treatment

MDA-MB-231 (breast cancer) and MCF-10A (nonmalignant breast epithelial) cells were obtained from the American Type Culture Collection (ATCC), (Rockville, MD, USA). The MDA-MB-231 cell line was cultured in Dulbecco’s modified Eagle’s medium (DMEM) (Biowest, Miami, FL, USA) supplemented with 2 mM L-glutamine, 100 U/mL penicillin, 100 µg/mL streptomycin and 5% fetal bovine serum (FBS) (Biowest, Miami, FL, USA). MCF-10A cells were cultured in DMEM-F12 (Biowest, Miami, FL, USA) supplemented with 100 U/mL penicillin, 100 µg/mL streptomycin, 10 µg/mL insulin (Biofluids, Rockville, MD, USA), 0.5 µg/mL hydrocortisone (Sigma-Aldrich, Toluca, State of México, México), 20 ng/mL epidermal growth factor (Upstate Biotechnology Incorporated, Lake Placid, NY, USA) and 5% FBS. The cells were maintained at 37 °C in a humidified 5% CO_2_–95% air atmosphere. For the treatment of cells with (-)-epicatechin, MDA-MB-231 and MCF10A cells were grown in phenol red-free media (Life Technologies, Gaithersburg, MD, USA) containing 5% FBS. (-)-Epicatechin was prepared in methanol, and the cells were treated with an inhibitory concentration of 50% (IC_50_ = 350 µM) as previously reported [27].

### 4.2. RNA Isolation and qRT–PCR

MDA-MB-231 and MCF-10A cells (5 × 10^5^ cells) that were untreated or treated with (-)-epicatechin were used to obtain total RNA. Cellular RNA was extracted using TRIzol™ Reagent (Invitrogen, Boston, MA, USA) according to the manufacturer’s instructions. The RNA obtained was dissolved in Milli-Q water and quantified at OD 260/280 in a NanoDrop™ 2000 c (Thermo Scientific, Boston, MA, USA). Twenty-five nanograms of RNA were subjected to a Verso 1-Step qRT–PCR Kit (Thermo Scientific, Boston, MA, USA) following the manufacturer’s protocol. The sequences of primers used were UCP2 forward: 5′-TCGGACACATAGTATGACCATTAG-3′; reverse: 5′-TCAGCAACAAGACGAGATAGAG-3′; and 18 s (internal control) forward: 5′-CCAGTAAGTGCGGGTCATAAG-3′; reverse: 5′-GGCCTCACTAAACCATCCAA-3′. cDNA synthesis and PCR were carried out in a PikoReal™ 24 Real-Time PCR System (Thermo Scientific, Boston, MA, USA). The conditions used were 50 °C for 15 min and 95 °C for 15 min, followed by 40 cycles at 95 °C for 15 s and 60 °C for 60 s. All the experiments were performed in triplicate, and the relative expression of the target genes was calculated using the 2^−ΔΔCq^ method with PikoReal™ Software 2.2 (Thermo Scientific, Boston, MA, USA).

### 4.3. Purification of UCP2 Protein and Western Blotting

UCP2 protein expression in mitochondria isolated via Derdak’s method was analyzed via Western blotting [51]. Briefly, MDA-MB-231 and MCF10A cells (5 × 10^6^ cells) that were untreated or treated with (-)-epicatechin were harvested with cold phosphate-buffered saline (PBS) containing 5 mM EDTA and centrifuged at 2500 rpm for 5 min. The resulting pellet was resuspended in isotonic mitochondrial buffer (210 mM mannitol, 70 mM sucrose, 1 mM EDTA, and 10 mM HEPES, pH of 7.5), supplemented with protease inhibitors (Complete Mini, EDTA-free protease inhibitor cocktail, Roche, (Sigma-Aldrich Química, S.A. de C.V., Toluca de Lerdo, Mexico) and homogenized. The homogenate was subsequently centrifuged at 600× *g* for 10 min at 4 °C. The supernatant was transferred to a new tube and centrifuged at 11,000× *g* for 15 min at 4 °C. The supernatant was discarded, and the pellet containing the mitochondria-enriched fraction was resuspended in extraction buffer (50 mM Tris-HCl, pH of 7.4, 1% sodium deoxycholate, 0.1% SDS, 150 mM NaCl, 1% Triton X-100) supplemented with protease inhibitors. The protein obtained was quantified by the Lowry method [52].

Western blotting was performed following standard procedures. Mitochondrial protein (50 µg) was electrophoresed on an SDS–polyacrylamide gel (10%) and transferred onto an Immun-Blot^®^ PVDF Membrane (Bio-Rad, Hercules, CA, USA). The blots were blocked with 5% nonfat dried milk in PBS. The membrane was subsequently incubated overnight with an anti-human *UCP2* antibody (Cell Signaling Technology, Danvers, MA, USA) in blocking buffer. After washing, the blot was incubated for 1 h with peroxidase-conjugated anti-mouse antibody (Bio-Rad) diluted in buffer. The blot was washed and developed via a chemiluminescence detection system (Clarity^TM^ Western ECL Substrate) (Bio-Rad). Bands were detected via an image analyzer, C-DiGit^®^ Blot Scanner (LI-COR^®^ Biosciences, Lincoln, NE, USA). Strep-tagged Precision Plus Protein All Blue Standards (Bio-Rad) were used as mass markers, which were visualized by chemiluminescence with Precision Protein StrepTactin-HRP conjugates (Bio-Rad). Densitometric analysis of the *UCP2* protein bands was performed to determine the amount of protein present using C-DiGit^®^ software (Image studio Ver 5.2.Ink) The β-tubulin protein was used as a loading control.

### 4.4. Cloning, Subcloning and Analysis of the Basal Promoter Activity of the Human UCP2 Gene

Genomic DNA from human white blood cells was used as a template for amplification via PCR of the 5′-region of the human *UCP2* gene (−2093/+297) using Phusion™ High-Fidelity DNA Polymerase (Thermo Scientific, USA). The primers used were designed for the UCP2 sequence (no. NG_011478.1, GenBank) as follows: forward: 5′-CAGTTTGTCCACAGGACATCTTATGA-3′; reverse: 5′-CTGGGAGACAGAGCGAAAGCC-3′. The size of the product was 2390 bp, and the PCR conditions were initial denaturation at 98 °C for 1 min and 35 cycles of 15 s at 98 °C, 15 s at 62 °C, and 2.5 min at 72 °C, with a final extension of 2 min at 72 °C. The PCR products were analyzed by agarose gel electrophoresis and purified (Supplementary Data S1A). The PCR product was cloned and inserted into the blunt end of the pJet1.2 vector using the CloneJET PCR Cloning Kit (Thermo Scientific) and sequenced (Supplementary Data S1B) by the BigDyeTM Terminator Cycle Sequencing Kits with Amplitaq DNA Polymerase on an ABI Prism Model 3F3 DNA Sequencer (Applied Biosystems, Foster, CA, USA). The PCR product (2390 bp) was extracted from the pJet1.2 vector, which was cut with the *Xba*I enzyme, filled with Klenow and cut with *Xho*I. Moreover, the pGLuc-Basic 2 vector (promoter-less Gaussia luciferase reporter gene) was cut with *Hind*III, filled with Klenow and cut with the *Xho*I enzyme for subcloning the PCR product (New England BioLabs^®^, Ipswich, MA, USA). This cloning generated the 2390-pGLuc construct (Figure 3A), and its activity was determined by transitory transfection of MDA-MB-231 and MCF10A cells (5 × 105 cells) with Lipofectamine™ 2000 (Invitrogen™, Thermo Scientific, Boston, MA, USA).

Luciferase activity was assayed in media with a BioLux^®^ Gaussia Luciferase Assay Kit (New England BioLabs^®^), and the luminescence signal was measured on a Synergy™ HT plate reader (BioTek^®^, Winooski, VT, USA). Luciferase activity was normalized to the pRL-SV40 luciferase activity of transfected cells processed with 100 µL of Luciferase Cell Lysis Buffer (New England BioLabs^®^) according to the manufacturer’s instructions.

### 4.5. Generation of UCP2 Promoter Deletion-Luciferase Fusion

The 2390 bp fragment cloned and inserted into the pGLuc vector was subsequently cut with restriction enzymes to generate different segments of the *UCP2* promoter. The constructs generated were 1697-pGLuc, 1083-pGLuc, 766-pGLuc, 483-pGLuc and 117-pGLuc. The construct 1620-pGLuc was generated from 2390-pGLuc with 770 bp deleted from the 5′ region. The constructs 483-pGLuc and 117-pGLuc were generated by PCR using specific primers for these regions and were subsequently cloned and inserted into the pGLuc vector. Finally, the constructs 100-pGLuc, 83-pGLuc and 51-pGLuc were generated from the construct 117-pGLuc with restriction enzymes. All the constructs generated were transfected into MDA-MB-231 and MCF10A cells, and the luciferase activity was analyzed in the absence and presence of (-)-epicatechin, as described below.

### 4.6. Transfection, (-)-Epicatechin Treatment and Promoter Activity

MDA-MB-231 and MCF10A cells (5 × 10^5^ cells) were plated onto 6-well plates and allowed to attach overnight. After washing twice with PBS, both cell lines were cotransfected with each of the constructs generated in pGLuc and *Renilla* luciferase plasmid (pRL-SV40) (Promega, Madison, WI, USA) as a control for transfection efficiency. The transfections were performed with Lipofectamine™ 2000 (Invitrogen™, USA) according to the manufacturer’s protocol. After 3 h of transfection, the media was changed to media without or with (-)-epicatechin (350 µM), and the cells were incubated for 48 h at 37 °C.

The promoter construct activity was determined through luciferase assays performed with media via a BioLux^®^ *Gaussia* Luciferase Assay Kit (New England BioLabs^®^). Luminescence signals were measured on a Synergy™ HT plate reader (BioTek^®^). The luciferase activity in the media was normalized to the luciferase activity detected in the transfected cells (pRL-SV40 plasmid). The cells were processed with 100 µL of Luciferase Cell Lysis Buffer (New England BioLabs^®^), and luciferase activity was determined following the manufacturer’s instructions.

### 4.7. Electrophoretic Mobility Shift Assays (EMSAs)

Nuclear proteins from MDA-MB-231 and MCF-10A cells were prepared as described by Luo et al. [53]. When the nuclear protein of cells treated with (-)-epicatechin was used, the cells were treated with the flavonoid (IC_50_ of 350 µM) for 48 h. Then, the cells were harvested, and the nuclear protein was obtained according to Luo et al. [53]. The protein concentration was determined by the Lowry method [52]. EMSAs were performed with a double-stranded oligonucleotide from the −82/+8 promoter region of the *UCP2* gene: forward, 5′-GGCAGGCCCGGCCCCGCCCCGCAGGCCCCACCCCGGGCCCCGCCCCCGAGGCTTAAGCCGCGCCGCCGCCTGCGCGGAGCCCCACTGCGA-3′; reverse, 5′-TCGCAGTGGGGCTCCGCGCAGGCGGCGGCGCGGCTTAAGCCTCGGGGGCGGGGCCCGGGGTGGGGCCTGCGGGGCGGGGCCGGGCCTGCC-3′. Oligonucleotides were end-labeled with IRDye^®^700 (LI-COR^®^ Biosciences). For binding studies, nuclear proteins (3 µg) were incubated with 25 fmol of labeled oligonucleotide in a 20 µL reaction volume containing 50 ng of salmon sperm DNA (Invitrogen™, USA), 12% glycerol, 20 mM HEPES, pH of 7.9, 100 mM KCl, 1 mM EDTA, and 1 mM DTT. The interaction mixture was incubated for 15 min at room temperature in the dark [54].

For the competition assays, a molar excess of the same unlabeled oligonucleotide (312.5,625, 937.5 and 1250 fmol) was included before the addition of the probe.

Supershift assays were carried out by preincubation overnight at 4 °C with 3 µg of nuclear protein with an anti-NRF1 polyclonal antibody (Genetex, Irvine, CA, USA) or a heterologous antibody before the addition of the labeled oligonucleotide. The complexes were resolved on a 4% nondenaturing polyacrylamide gel. The gel was scanned at 700 nm and analyzed with an Odyssey^®^ Infrared Imaging System (LI-COR^®^ Biosciences).

### 4.8. Bioinformatic Analysis of the Promoter Region (−82/+8) of the UCP2 Gene

In silico analysis was performed to localize putative transcription factor-binding sites of +8 to −82 in the 5′ region of the *UCP2* gene. We used MatInspector to determine possible regulatory sites inside the promoter region of the *UCP2* gene [55]. Potential sites for transcription factors were selected on the basis of 0.85 core similarity.

### 4.9. Statistical Analysis

All the statistical analyses were performed with GraphPad^®^ Prism 5.01 using two-tailed unpaired Student’s t-tests. The significant differences are presented as the means ± standard deviations of three independent experiments, where *p*-values less than 0.05 were considered statistically significant.

## Data Availability

All data are available in the text and in Supplementary Files.

## Supplementary Materials

The following supporting information can be downloaded at https://www.mdpi.com/article/10.3390/ijms26094102/s1.
