# Supplementary material for: Epicatechin Decreases UCP2 Gene Expression in MDA-MB-231 Breast Cancer Cells by the Presence of a Regulatory Element in the Promoter
Source: Int J Mol Sci. 2025 Apr 25;26(9):4102. doi: 10.3390/ijms26094102 (PMC12071687; doi:10.3390/ijms26094102)
Supplement: Supplementary file 1 [file ijms-26-04102-s001.zip › Suplplementary Data S2.pdf]

Table S1. Transcription factors predicted by MatInspector analysis in aligned regions

Matrix Family	Family Information	Start position (5´)	Core position (5´-3´)	End position (3´)	Core similarity	Matrix similarity	Sequence
V$OAZF	Olfactory associated zinc finger protein	-102	-100 to -97	-86	1	0.742	cc**GCAC**ccaa
V$ZTRE	Zinc transcriptional regulatory element	-71	-69 to -66	-55	0.863	0.953	cc**CGCC**ccgc
V$NRF1	Nuclear respiratory factor 1 (NRF1)	-69	-64 to -61	-53	1	0.81	cc**CGCA**ggcc
V$ZF02	C2H2 zinc finger transcription factors	-63	-58 to -55	-41	1	1	agg**CCCC**acc
V$ZTRE	Zinc transcriptional regulatory element	-57	-49 to -46	-41	0.879	0.772	cc**GGGC**cccg
V$GCF2	Transcriptional repressor GC-binding factor 2	-56	-53 to -50	-38	1	0.937	cca**CCCC**ggg
V$HOMF	Homeodomain transcription factors	-41	-32 to -29	-23	1	0.895	gg**CTTA**agcc
V$NRF1	Nuclear respiratory factor 1 (NRF1)	-28	-24 to -21	-12	1	0.833	agcc**GCGC**cgc
O$TF2B	RNA polymerase II transcription factor II B	-26	-23 to -20	-20	1	1	ccg**CGCC**
V$ZF15	C2H2 zinc finger transcription factors	-22	-13 to -10	-6	1	0.898	gcc**TGCG**cgg

Matrix (groups of functionally similar transcription factors), starting/core position/ending core; core similarity; matrix similarity factor (0–1); elements.
